# Familial Hemophagocytic Lymphohistiocytosis with A665G Perforin Gene Mutation: A Case Report

**DOI:** 10.5505/tjh.2012.62134

**Published:** 2012-10-05

**Authors:** İdil Yenicesu, De Saint Basile Geneviève, Hamdi Cihan Emeksiz, Buket Dalgıç

**Affiliations:** 1 Gazi University, School of Medicine, Department of Pediatrics, Ankara, Turkey; 2 Hôpital Necker-Enfants Malades, Paris, France

**Keywords:** Familial hemophagocytic lymphohistiocytosis, Perforin gene, A665G homozygous mutation, Prenatal diagnosis, Turkey

## Abstract

Familial hemophagocytic lymphohistiocytosis (FHL) is a genetically heterogeneous disease. Presentation of the disease such as primarily fever, hepatosplenomegaly, and cytopenia, which are the results of functional degradation in cytotoxic T-lymphocytes and natural killer cells, activation of macrophages and T-lymphocytes, over production of proinflammatory cytokines, and hemophagocytosis. In all, 5 genetic loci have been identified in FHL, and all known affected genes encode critical components of the granule exocytosis pathway, which is essential for the release of cytotoxic granules and proteases that are necessary for targeted cell death. Herein we present an FHL patient with a severe clinical course and a very rare perforin gene mutation. The patient was homozygous for A665G mutation. However, the child died in a short period of time. Prenatal diagnosis was performed in the family and the fetus was found to be heterozygous for the mutation.

## Abstract (tr)

Ailesel Hemofagositik Lenfohistiyositozis(AHL), genetik olarak oldukça heterojen bir hastalıktır. Sıklıkla ateş, hepatosplenomegali, sitopeni gibi bulgular görülebilir. Bu bulgular doğal katil hücreler ve sitotoksik T lenfositlerde fonksiyonel bozulma, makrofaj ve T lenfositlerde aktivasyon, proinflamatuar sitokinlerde artış ve hemofagositoz sonucu oluşur. AHL’de 5 ayrı genetik bölge tanımlanmıştır. Tüm bu bölgelerin ortak özelliği granul ekzositoz yolağı için kritik görev gören maddelerin yapımında rol oynamalarıdır. Bu makalede çok nadir perforin mutasyonuna sahip ve ağır klinik tabloyla erken dönemde kaybedilen bir olgu sunulmuştur. Hastada homozigot A665G mutasyonu bulundu. Hasta kısa sure sonra kaybedildi. Sonraki gebelikte prenatal tanı konuldu. Fetusda aynı mutasyonun heterozigotluğu gösterildi.

## INTRODUCTION

Familial hemophagocytic lymphohistiocytosis (FHL) is a genetically heterogeneous disease. The clinical presentation of FHL includes primarily fever, hepatomegaly, and cytopenia, which are the results of functional degradation in cytotoxic T-lymphocytes and natural killer (NK)-cells, activation of macrophages and T-lymphocytes, excessive production of proinflammatory cytokines, and hemophagocytosis [1,2,3]. According to a study from Sweden, the incidence of FHL is 1.2 in 1,000,000 [4], but as intermarriage is common in Turkey the incidence of FLH in Turkey is probably higher. A study from Turkey reported that the frequency of primary hemophagocytic lymphohistiocytosis (HLH) is 7.5 in 10,000 among hospitalized patients [5].

Five loci causing FHL have been identified, as well as the underlying genetic defect for 4 of them. FHL1 is a locus first described in 1999 following linkage analysis in 2 Pakistani families, although the gene remains unknown. FHL2 encodes mutations in the perforin (PFR1) gene. Mutations in the UNC13D gene (FHL3) interfere with the role of the encoded protein Munc 13-4 in cytolytic granule exocytosis and FHL4 states the mutations in the STX11 gene and production of syntaxin 11, which also plays a role in cytotoxic granule release. Recently, mutations in STXB2, which encode syntaxin binding protein 2 (Munc 18-2) and cause impaired protein expression and impaired NK-cell cytotoxic granule exocytosis, were also described (FHL5) [6,7,8,9,10,11,12]. Perforin gene mutations are not uncommon in Turkey. Herein we present an FHL patient with a severe clinical course and a very rare homozygous perforin gene mutation.

## CASE REPORT

A 3.5-month-old male infant presented with abdominal protuberance, fever, and cough, and was admitted to hospital. One week earlier he developed a fever with cough, was diagnosed as lower respiratory tract infection, and was given antibiotic treatment at another hospital. The parents are first-degree cousins. The initial physical examination performed at our hospital showed the following signs: jaundice, grunting, intercostal retraction, and marked hepatosplenomegaly. No one else in the patient’s family had a similar history. 

Complete blood count findings were as follows: white blood cell count: 4.7x10^9^ L^–1^; absolute neutrophil count: 752 mm^–3^; hemoglobin: 4.8 g dL^–1^; platelet count: 15 x 10^9^ L^–1^. Further laboratory test results include elevated transaminases (ALT: 4202 u L^–1^; AST: 1689 u L^–1^), elevated lactate dehydrogenase (1984 u L^–1^), and elevated serum ferritin (40,000 μg mL^–1^). Serum triglycerides was 207 mg mL^–1^, serum total bilirubin was 6.14 mg dL^–1^, serum direct bilirubin was 4.78 mg dL^–1^, serum uric acid was 30.9 mg dL^–1^, and fibrinogen was 50 mg dL^–1^. Arterial blood gas analysis was consistent with metabolic acidosis. Due to respiratory depression the patient was mechanically ventilated with multi-component transfusion support. He was given antibiotic treatment (teicoplanin, ceftriaxone, and fluconazole) during his hospitalization. 

This patient underwent bone marrow aspiration, which showed profuse hemophagocytosis. The clinical and laboratory findings, and consanguineous parents strongly suggested FLH which lead to the commencement of the HLH 2004 protocol [13]. The patient’s elevated uric acid level and acidosis were treatment resistant, which led to the decision to perform peritoneal dialysis. Peritoneal dialysis does not have a clinically significant effect on cyclosporine, etoposide, or dexamethasone plasma clearance. As such, supplemental doses were not given during the dialysis procedure. Despite all treatment efforts, the patient died 30 h after admission due to multi-organ failure. 

Postmortem splenic examination showed profuse hemophagocytosis. While alive the patient was in extremely poor clinical condition and was intubated; therefore, investigation of the central nervous system could not be performed, but postmortem cerebrospinal fluid examination showed mononuclear cell infiltration. Written informed consent for abdominal autopsy was obtained from the patient’s family. The bone marrow core biopsy showed normal cellular components and abundant hemophagocytic macrophage infiltration based on CD68 immunohistochemical staining. Liver smear showed extramedullary hematopoiesis and microvesicular steatosis, as well as massive hemophagocytic macrophage infiltration via CD68, particularly in portal regions and in some sinusoidal regions. An increase in sinusoidal macrophages was noted in lymph nodes dissected from the peripancreatic region and mesocolon. Adrenal medulla showed massive CD68-positive macrophage infiltration. Focal histiocytic cellular groups were observed in kidney specimens. 

Post-mortem genetic testing showed homozygosity for A665G PRF 1 mutation. The patient was the parents’ first child—the result of the mother’s first pregnancy. As the parents were consanguineous, the family was provided genetic counseling about subsequent pregnancies. One year later the mother was again pregnant and the fetus was determined to be a carrier for the same mutation; however, spontaneous abortion terminated the pregnancy.

## DISCUSSION

When the immune system is triggered by an infectious agent histiocytes (macrophages and dendritic cells), NKcells, and cytotoxic T-lymphocytes (CTLs) are activated, mutually stimulating each other via receptor interaction, as well as secretion of a variety of inflammatory cytokines and chemokines. In immune-competent individuals this concerted action leads to of the death of infected cells, removal of antigen, and termination of the immune response. In cases of inherited or acquired NK-cell and CTL defects this process is impaired, infected cells do not undergo apoptosis, and persistently high cytokine levels lead to the clinical picture of HLH [2].

The cytotoxic function of NK-cells and CTLs is mediated by release of cytolytic granules, which contain perforin, granzymes, and other serine-specific proteases, into the immunological synapse that forms between the effector and the target cell. Cytolytic granules must travel to the contact site, dock and fuse with the plasma membrane, and release their contents [2]. All known affected genes encode critical components of the granule exocytosis pathway, which is essential for the release of cytotoxic granules and proteases necessary for targeted cell death in HLH [1]. The most common mutations involve the perforin 1 gene, as in the presented case [6,14]. Perforin is expressed in the granules of T-cells and NK-cells, and creates pores in the cytoplasm of targeted cells, thereby allowing granzymes and proteases to enter cells and ultimately induce apoptosis after entering the cell nucleus [1]; however, the perforin gene mutation may not lead to the development of the clinical features of FHL until a patient comes into contact with viral infectious agents and environmental agents [12,15].

Lee et al. previously described a case where one allel has the same mutation as our case while the mutation of the other allel could not be found [16]. Recently, the same mutation in homozygous state was described in a family from Turkey [17]. To the best of our knowledge the presented case is the second homozygous case with this mutation in the current literature. Lee et al. showed that this mutation occurs in a region of the perforin protein that exhibits amphipathic conformation in order to interact with and traverse the membrane lipid bilayer, and exhibits the highest degree of homology to the putative lipid-binding domain of the complement components. The absence of NK-cell function in their patient suggests that mutation in this region interferes with the function of the perforin protein [16].

In addition to the diagnosis of FHL, an understanding of its genetic or acquired nature is also of great value, especially in families with consanguineous marriage and a history of early onset of FHL in their offspring. The family of the presented case had the same characteristics. Prenatal diagnosis was of special importance to the presented case’s family because he was their first child and they planned to have another baby. Currently, the most important problem is variation in FHL mutations according to country— almost 50% of which have not yet been determined. This was reported to be as 50% in Turkey and 70% in Germany [13].

Due to the fact that FHL is quite common in Turkey, identification of the molecular pathologies in FHL patients is critical. Okur reported that 9 of 37 Turkish FHL patients (24%) were FHL type-2 based on linkage analysis. Genomic sequencing of the entire coding region of the perforin gene led to the identification of 5 different pathologic changes in these patients. In all, 3 patients (33%) carried the same nonsense W374X mutation in exon 3, and the 6 remaining patients (67%) had 4 different missense variations (G149S, V50M, A91V, and novel A523D). Among these missense changes, G149S S was detected in 2 patients, V50M in 1 patient, and A91V in 2 patients [18]. In contrast, 2 earlier studies reported that 30% and 44% of Turkish FHL patients had perforin mutations, of which, 67% and 86%, respectively, had W374X mutation [10,14]. These 3 studies indicate that the incidence of perforin mutation is similar, ranging from 24%-44% and that W374X mutation is the most common type of perforin mutation in the Turkish population [10,14,18].

In the Turkish population perforin gene mutation is the most commonly detected mutation in the 4 genes known to be responsible for HLH. Among perforin gene mutations, W374X is known as the Turkish mutation and is common in Turkey; this mutation, which has a severe clinical outcome, is thought to have first emerged in Southeast Anatolia [19]. Clinical symptoms appear at birth and infants are usually symptomatic until 6 months of age. The A665G (H222R) mutation observed in the presented case is rare and associated with a severe disease course. It may occasionally lead to hydrops fetalis or become symptomatic during the first months of life [19,20]. Risma et al. classified perforin gene mutations into 3 groups, based on flow cytometry, immunohistochemistry, and immunoblotting methods: class 1 are missense mutations with partial maturation of perforin; class 2 are missense mutations with no apparent proteolytic maturation of perforin; class 3 are missense mutations with no recognizable forms of perforin. Among these 3 classes, class 3 missense mutations are associated with the most severe disease course [21]. The A665G mutation in the presented case was in class 3 and was associated with severe clinical manifestations, resulting in mortality at age 3 months.

The aim of this case presentation was to increase pediatricians’ awareness of HLH. HLH is a life-threatening disease that may be difficult to distinguish from severe sepsis. A simple clinical approach may be helpful in diagnosing HLH. As such, autopsy studies suggest that HLH may be under recognized in intensive care unit patients [22,23,24]. Several diagnostic criteria protocols have recently been developed for HLH. The most current (HLH-2004 protocol) suggests that HLH can be diagnosed if 5 of 8 diagnostic criteria are met [13]. In recent years elevated levels of ferritin have become more important in diagnosis, especially levels >10,000 mg dL^–1^ [25]. The presented case had an extremely high ferritin level (40,000 mg dL^–1^). HLH should be ruled out in any previously healthy child that abruptly develops fever, hepatosplenomegaly, cytopenia, and jaundice. 

Chemo-immunotherapy based on the HLH-2004 protocol includes corticosteroids, epipodophyllotoxins, and cyclosporine, and succeeds in controlling the disease in the majority of patients; however, cure is rarely achieved. Most patients suffer an early death unless they undergo hematopoietic stem cell transplantation (HSCT), which is the only curative approach available to date [13]. In conclusion, mutations like V50M in HLH patients become symptomatic later in life [18] and are associated with a slow clinical course, whereas mutations like W374X and A665G are associated with early onset of clinical symptoms, a rapid and severe disease course, and mortality shortly afterward due to multiple organ failure, as in the presented case. As such, the clinical outcome in HLH patients often depends on how rapidly and precisely diagnosis is established. In the near future it is expected that other candidate genes will be identified that will facilitate the establishment of appropriate individualized therapies, including chemotherapy, immunotherapy, HSCT, and perhaps gene therapy. Until that time, as practicing clinicians our task is to acquire sufficient knowledge for timely diagnosis and treatment of HLH.

**Conflict of Interest Statement **

The authors of this paper have no conflicts of interest, including specific financial interests, relationships, and/ or affiliations relevant to the subject matter or materials included.

## References

[1] Verbsky JW, Grossman WJ (2006). Hemophagocytic lymphohistiocytosis: Diagnosis, pathophysiology, treatment and future perspectives. Ann Med.

[2] Janka GE (2007). Familial and acquired hemophagocytic lymphohistiocytosis. Eur J Pediatr.

[3] Janka GE (2007). Hemophagocytic syndromes. Blood Reviews.

[4] Henter JI, Elinder G, Söder O, Ost A (1991). Incidence in Sweden and clinical features of familial hemophagocytic lymphohistiocytosis. Acta Paediatr Scand.

[5] Gürgey A, Göğüş S, Ozyürek E, Aslan D, Gümrük F, Cetin M, Yüce A, Ceyhan M, Seçmeer G, Yetgin S, Hiçsönmez G (2003). Primary hemophagocytic lymphohistiocytosis in Turkish children. Pediatr Hematol Oncol.

[6] Stepp SE, Dufourcq-Lagelouse R, Le Deist F, Bhawan S, Certain S, Mathew PA, Henter JI, Bennett M, Fischer A, Kumar V (1999). Perforin gene defects in familial hemophagocytic lymphohistiocytosis. Science.

[7] Ohadi M, Lalloz MR, Sham P, Zhao J, Dearlove AM, Shiach C, Kinsey S, Rhodes M, Layton DM (1999). Localization of a gene for familial hemophagocytic lymphohistiocytosis at chromosome 9q21.3-22 by homozygosity mapping. Am J Hum Genet.

[8] Feldmann J, Callebaut I, Rapuoso G, Certain S, Bacq D, Dumont C, Lambert N, Ouachee-Chardin M, Chedeville G, Tamary H, Minard-Colin V, Vilmer E, Blanche S, Le Deist F, Fischer A (2003). Munc 13-4 is essential for cytolytic granules fusion and is mutated in a form of familial hemophagocytic lymphohistiocytosis(FLH3). Cell.

[9] Zur Stadt U, Schmidt S, Kasper B, Beutel K, Diler AS, Henter JI, Kabisch H, Schneppenheim R, Nurnberg P, Janka G, Hennies HC (2005). Linkage of familial hemophagocytic lymphohistiocytosis (FHL) type-4 to chromosome 6q24 and identification of mutations in syntaxin 11. Hum Mol Genet.

[10] Zur Stadt U, Beutel K, Kolberg S, Schneppenheim R, Kabisch H, Janka G, Hennies HC (2006). Mutation spectrum in children with primary hemophagocytic lymphohistiocytosis: Molecular and functional analyses of PRF1, UNC13D, STX11 and RAB27A. Hum Mutat.

[11] Côte M, Ménager MM, Burgess A, Mahlaoui N, Picard C, Schaffner C, Al-Manjomi F, Al-Harbi M, Alangari A, Le Deist F, Gennery AR, Prince N, Cariou A, Nitschke P, Blank U, El-Ghazali G, Ménasché G, Latour S, Fischer A (2009). Munc18-2 deficiency causes familial hemophagocytic lymphohistiocytosis type 5 and impairs cytotoxic granule exocytosis in patient NK cells. J Clin Invest.

[12] Freeman HR, Ramanan AV (2011). Review of haemophagocytic lymphohistiocytosis. Arch Dis Child.

[13] Henter JI, Horne AC, Arico M, Egeler RM, Filipovich AH, Imashuku S, Ladish S, McClain K, Webb D, Winiarski J, Janka G (2007). HLH-2004: Diagnostic and therapeutic guidelines for hemophagocytic lymphohistiocytosis. Pediatr Blood Cancer.

[14] Göransdotter E, Fadeel B, Nilsson-Ardnor S, Söderhäll C, Samuelsson A, Janka G, Schneider M, Gurgey A, Yalman N, Révész T, Egeler R, Jahnukainen K, Storm-Mathiesen I, Haraldsson A, Poole J, Nordenskjöld M, Henter J (2001). Spectrum of perforin gene mutations in familial hemophagocytic lymphohistiocytosis. Am J Hum Genet.

[15] Albayrak M, Kaya Z, Yılmaz-Keskin E, Stadt UZ, Koçak U, Gürsel T (2009). Fatal Epstein-Barr virus infection in a case familial hemophagocytic lymphohistiocytosis with syntaxin-11 mutation. Turk J Pediatr.

[16] Lee SM, Villanueva J, Sumegi J, Zhang K, Kogawa K, Davis J, Filipovich AH (2004). Characterisation of diverse PRF1 mutations leading to decreased natural killer cell activity in North American families with haemophagocytic lymphohistiocytosis. J Med Genet.

[17] Aslan D (2011). A665G mutation in PRF1 in a Turkish infant with familial hemophagocytic lymphohistiocytosis. Pediatr Blood Cancer.

[18] Okur H, Balta G, Akarsu N, Oner A, Patiroglu T, Bay A, Sayli T, Unal S, Gurgey A (2008). Clinical and molecular aspects of Turkish familial hemophagocytic lymphohistiocytosis patients with perforin mutations. Leukemia Research.

[19] Balta G, Okur H, Unal S, Yarali N, Gunes AM, Unal S, Turker M, Guler E, Ertem M, Albayrak M, Patiroglu T, Gurgey A (2010). Assessment of clinical and laboratory presentations of familial hemophagocytic lymphohistiocytosis patients with homozygous W374X mutation. Leuk Res.

[20] Lipton JM, Weastra S, Hawerty CE, Roberts D, Harris NL (2004). Case records of the Massachusetts General Hospital. Weekly clinicopathological exercises. Case 28-2004. Newborn twins with thrombocytopenia, coagulation defects, and hepatosplenomegaly. N Engl J Med.

[21] Risma KA, Frayer RW, Filibovich AH, Sumegi J (2006). Aberant maturation of mutant Perforin underlies the clinical diversity of hemophagocytic lymphohistiocytosis. J Clin Invest.

[22] Gauvin F, Toledano B, Champagne J, Lacroix J (2000). Reactive hemophagocytic syndrome presenting as a component of multiple organ dysfunction syndrome. Crit Care Med.

[23] Strauss R, Neureiter D, Westenburger B, Wehler M, Kirchner T, Hahn EG (2004). Multifactorial risk analysis of bone marrow histiocytic hyperplasia with hemophagocytosis: A postmortem clinicopathologic analysis. Crit Care Med.

[24] Nahum E, Ben-Ari J, Stain J, Schonfeld T (2000). Hemophagocytic lymphohistiocytic syndrome: Unrecognized cause of multiple organ failure. Pediatr Crit Care Med.

[25] Allen CE, Yu X, Kozinetz CA, Mc Clain KL (2008). Highly elevated ferritin levels and the diagnosis of hemophagocytic lymphohistiocytosis. Pediatr Blood Cancer.

